# International Survey of Current Approaches to the Management of Neuropathic Corneal Pain by Experts

**DOI:** 10.1007/s40123-025-01242-8

**Published:** 2025-10-08

**Authors:** Samy El Omda, Nikolaos Tzoumas, Margarita Calonge, Francisco Figueiredo

**Affiliations:** 1 Department of Ophthalmology, Royal Victoria Infirmary, Newcastle upon Tyne, UK; 2 Biosciences Institute, Medical School, Newcastle University Newcastle Upon Tyne, Newcastle upon Tyne, UK; 3 Instituto de Oftalmobiologia Aplicada (IOBA), Universidad de Valladolid, Valladolid, Spain

**Keywords:** Cornea/innervation, Corneal diseases/diagnosis, Cornea/pain, Eye pain/etiology, Eye pain/diagnosis, Eye pain/therapy, Neuropathic pain

## Abstract

**Introduction:**

Neuropathic corneal pain (NCP) is a challenging condition with limited consensus on its diagnosis and management. This study aimed to gather global insights from corneal specialists on the causes, investigative approaches, and management strategies for NCP.

**Methods:**

A 32-question survey covering demographic, causes, investigations, treatments, and multidisciplinary engagement was sent to 152 invited international corneal specialists; 51 (34%) responded. We explored descriptive statistics and examined how responder characteristics influenced their answers.

**Results:**

The most reported causes of NCP were chronic ocular surface disease (*n* = 41; 41%) and post-surgical factors (*n* = 34; 34%). The most common investigations, routinely performed by respondents, were the anesthetic challenge test, Schirmer’s test, and corneal esthesiometry. In vivo confocal microscopy (IVCM) was routinely used by 37% (*n* = 19), with 69% (*n* = 29) of specialists stating that an abnormal result influenced their management. Ocular surface and pain questionnaires were used by 69% (*n* = 35), with the Ocular Surface Disease Index being the most popular (*n* = 25; 31%). Common treatments included artificial tears (*n* = 48; 94%), serum/plasma-derived tears (*n* = 41; 80%), topical corticosteroids (*n* = 34; 67%), and topical cyclosporin (*n* = 30; 59%). Only 38% (*n* = 19) felt comfortable independently prescribing systemic pharmacotherapy. A multidisciplinary approach was adopted by 47% (*n* = 24), with the two most common specialties involved being pain management (*n* = 30; 37%) and neurology (*n* = 26, 32%).

**Conclusions:**

This survey provides valuable global insights into the causes, investigations, and management of NCP from the perspective of corneal specialists. These findings support further research and the development of guidelines to address this challenging condition.

## Plain Language Summary

Neuropathic corneal pain is a challenging condition due to patients reporting very significant eye pain even when there is no obvious problem on the eye’s surface when examined. This study asked eye doctors, specifically corneal pain specialists, worldwide about the causes of neuropathic corneal pain, their investigative methods, and their treatment approaches. We sent a survey with 32 questions to 152 corneal specialists, and 51 replied. Most thought long-term problems with the eye surface or surgery on the eye caused the condition. The most commonly used tests involved seeing if the pain reduces with numbing drops, checking for dry eye, and checking how sensitive the nerves at the front of the eye were. Only about a third used a special microscope test called in vivo confocal microscopy, but when it showed nerve problems, this often changed how they treat patients. Doctors most often used treatments like lubricants, blood-derived eye drops (i.e., serum or plasma), steroid drops, and cyclosporin drops. However, few felt confident prescribing oral medicines that affect the whole body to treat the eye pain. Almost half worked with other specialists to manage the condition, especially pain and brain doctors. These results show there are different ways to investigate and treat neuropathic corneal pain and highlight the need for clearer guidelines to help doctors care for people living with this painful condition.

## Key Summary Points

**Table:** 

*Why carry out this study?*
Neuropathic corneal pain (NCP) is a relatively new and vaguely defined condition in ophthalmology, which presents significant management challenges due to its complex diagnosis and limited treatment options.
This study highlights the current practice patterns of international experts in identifying and managing NCP.
*What was learned from the study?*
NCP is most often associated with chronic ocular surface disease and post-surgical factors.
The current mainstay of pharmacological management is topical agents, with systemic agents being used only cautiously.
It is recommended to manage NCP using a multidisciplinary approach, as well as utilizing questionnaires to help assess the impact of NCP on quality of life and evaluate treatment response and disease progression.

## Introduction

Neuropathic pain originates from lesions or diseases affecting the somatosensory nervous system [1]. Neuropathic corneal pain (NCP)—also known as neuropathic ocular pain, neuropathic eye pain, corneal neuropathic pain, ocular pain syndrome, keratoneuralgia, phantom cornea, pain without stain and corneal allodynia—is characterized by corneal pain in response to normally non-painful stimuli, stemming from an oversensitive neuronal network in the cornea due to peripheral neuronal damage or disturbances in central pain pathways [2].

As a relatively new and vaguely defined condition in ophthalmology, NCP presents significant management challenges due to its complex diagnosis and limited treatment options [3, 4]. Patients typically experience a triad of hyperalgesia, allodynia, and spontaneous pain, yet often show minimal physical signs during slit-lamp examinations [4]. This discrepancy has led to suspicions of malingering or psychosomatic disorders among patients with NCP, resulting in the moniker “pain without stain”. Consequently, these patients are often dismissed or misdiagnosed with isolated dry eye disease [5], overlooking underlying causes that may require tailored therapeutic approaches [3].

The diagnostic complexity and lack of clinical consensus hinder the initiation of target clinical treatment, causing patients considerable distress [4]. Previous recommendations suggest supplementing routine slit-lamp examination with functional somatosensory testing (e.g., anesthetic challenge test, corneal esthesiometry), symptom severity questionnaires, and in vivo confocal microscopy (IVCM) [3]. Given these uncertainties, there is an urgent need for consensus guidelines to standardize practices. This survey aims to map the current global state of NCP diagnosis, testing, and management, to help identify research priorities, and lay the foundation for the development of clinical guidelines to improve patient outcomes.

## Methods

### Ethics Approval

This study was approved by the Research Policy Intelligence and Ethics Team at Newcastle University—Ref: 52,149/2023, who judged the project low-risk and waived formal ethics review. Informed consent was obtained from all participants. Patients and the public were not involved in the design and conduct of this study.

### Survey Design

An online survey was designed and delivered to clinical academic professionals with significant experience in managing NCP. We surveyed respondents’ practice, including preferred investigations, management, and multidisciplinary engagement. Questions were based on clinical experience and current literature on the condition [3, 5]. Several iterations were made before the survey was finalized through the expert advice of authors MC and FF. The survey consisted of 32 questions, including six demographic questions, one on causes, ten investigation questions, ten treatment questions, and five on multidisciplinary engagement. A copy of the survey is provided in the supplemental data.

### Survey Responses

Responses to all questions were mandatory. Conditional queries were used to tailor follow-up questions to respondents’ earlier responses. The survey contained multiple-choice questions, free-text boxes, and Likert-type scales. Responses were anonymized.

### Survey Administration

One hundred and seventeen professionals were identified through our senior author’s network, and 35 professionals were identified through a PubMed search in November 2022, in which senior authors of relevant papers on neuropathic corneal disease and refractive surgery were recorded. Questionnaires were distributed via e-mail to identified specialists worldwide in May 2023, with an additional two reminders sent between May and June 2023. Responses were received from 52 (34%) of the 152 professionals invited. Given the clinical focus of many of the survey’s questions, respondents with no clinical practice (*n* = 1, a university professor) were excluded from the final results, leaving 51 responses. This explains the discrepancy between these results and our previously published abstract [6].

### Data Analysis

Data were analyzed using R (Version 4.3.1, The R Foundation, Vienna, Austria). Descriptive statistics, including distributions and measures of central tendency, were calculated. For free-text questions, content analysis was used to identify sub-categories, which were then analyzed. The Wilcoxon rank-sum test was used to identify any differences in answers from responses from the United States and Europe.

## Results

### Demographic of Respondents

Of the 51 respondents, 37 (73%) were employed by an academic/University hospital, with six (12%) employed by a private hospital, three (6%) in independent practice, three (6%) working in private and academic centers, and two (4%) retired ophthalmologists. Eighteen of 51 (35%) respondents practiced in North America, 25 of 51 (49%) in Europe, and the final eight (16%) in other regions, i.e., Africa, Asia, and Australia. Forty-three (84%) had been practicing for over 15 years post-residency, four (8%) had between 11 and 15 years’ experience, and four (8%) had between 6 and 10 years of experience. None had been in practice for 5 years or less. Overall, responses were received from 18 different countries.

### Causes and Encounters of NCP

Twenty-one of 51 (41%) of respondents encountered more than 15 cases of NCP per year, with 11 of 51 (22%) encountering 11 to 15 cases, eight of 51 (16%) encountering six to ten cases, and 11 of 51 (22%) encountering up to five cases per year. We asked our respondents to state the top two most common causes of NCP encountered in their experience. The most popular response was chronic ocular surface disease, with 41 of 101 causes mentioned (41%), followed by post-surgical (34 of 101; 34%). No other cause had a greater than 10% response rate, but they included infections (8%), miscellaneous (trigeminal neuralgia, fibromyalgia) (8%), systemic neuropathies (6%), and toxic keratopathy (2%). We also note three reports of “Primary” causes where there was no background co-pathology identified.

### Investigations for NCP

**Fig. 1:**
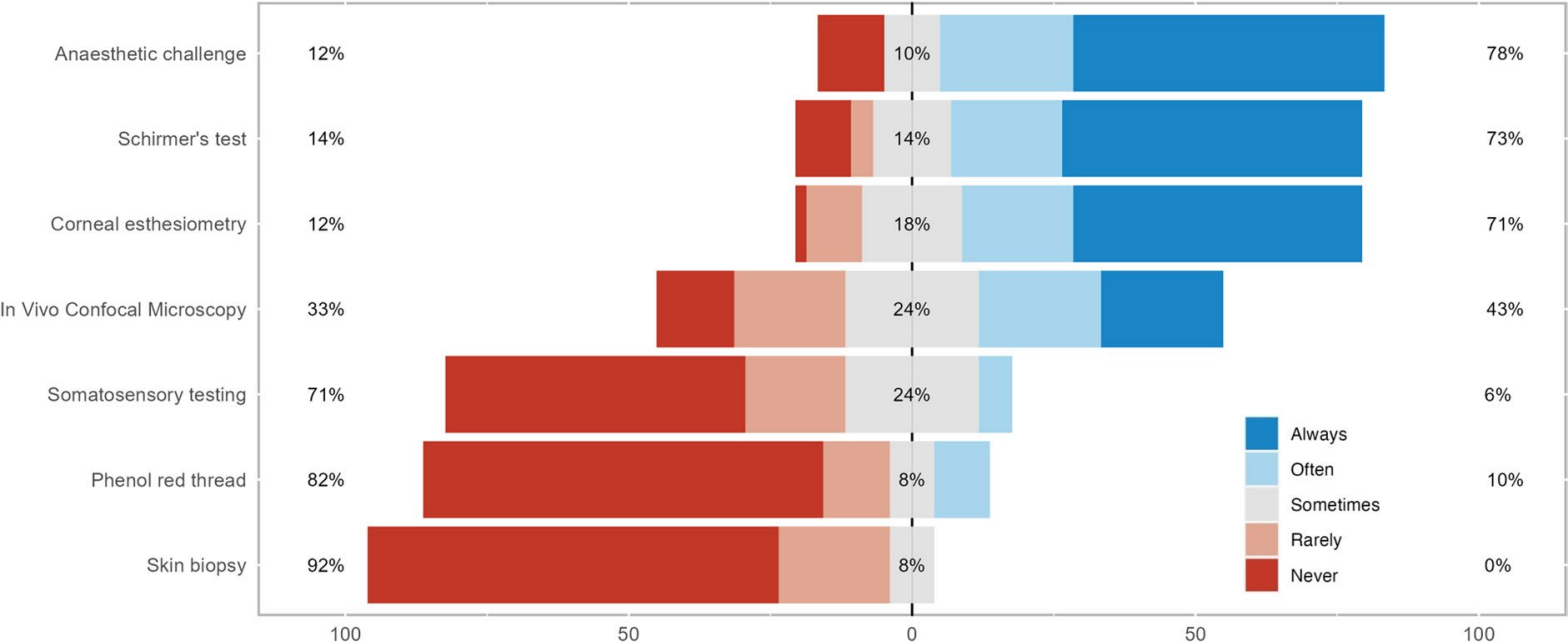
Stacked bar chart of the investigations used for neuropathic corneal pain

Three investigations were always used by most respondents: anesthetic challenge test, Schirmer’s test, and corneal esthesiometry (Fig. 1).

#### IVCM

Of the 51 clinicians, 19 (37%) routinely used IVCM in suspected NCP, 20 (39%) would use IVCM but only when examination findings are minimal and clinical suspicion is high, with 12 (24%) not using IVCM routinely.

Those who do not use IVCM routinely were asked to state the reasons why. Nine of 16 (56%) reported this was due to the unavailability of equipment or trained staff, four of 16 (25%) stated it will not alter the diagnosis or treatment plan, two of 16 (13%) did not find it specific enough of a test, with one of 16 (6%) reporting time constraints.

Of those who do perform IVCM, 29 of 42 (69%) stated an abnormal IVCM would influence their management in clinically suspicious cases of NCP with 13 of 42 (31%) saying it would not influence their management. These 29 respondents were asked to state what findings would influence their management in a free-text box. These were analyzed and organized into five categories “other nerve abnormalities” (*n* = 16, 34%), “micro-neuromas” (*n* = 15, 32%), “inflammatory changes (including dendritic cells)” (*n* = 14, 30%), and “damage to subepithelial plexus” (*n* = 2, 4%).

#### Ocular Surface and Pain questionnaires

Thirty-five of 51 respondents (69%) reported routinely using ocular surface and pain questionnaires in the assessment of NCP. The Ocular Surface Disease Index (OSDI) was the most commonly used questionnaire (*n* = 25, 31%), followed by the Ocular Pain Assessment Survey (OPAS) (*n* = 15, 19%), and the 5-Item Dry Eye Questionnaire (DEQ-5) (*n* = 14, 17%). The Neuropathic Pain Symptom Inventory (NPSI) was only used by three respondents, with the modified NPSI-eye being used by five. Twenty-five of 51 (49%) used such questionnaires at the initial presentation, 16 of 51 (31%) clinicians used it at regular intervals, and 18 of 51 (35%) used them only dynamically, in response to changing symptoms or treatments. Among 16 of 51 respondents who did not routinely use questionnaires, the main reasons were the lack of available questionnaires or trained staff to administer these (*n* = 9), or a lack of time (*n* = 4). Others believed that such tools would not alter the diagnosis or treatment plan (*n* = 5).

### Treatments

**Fig. 2:**
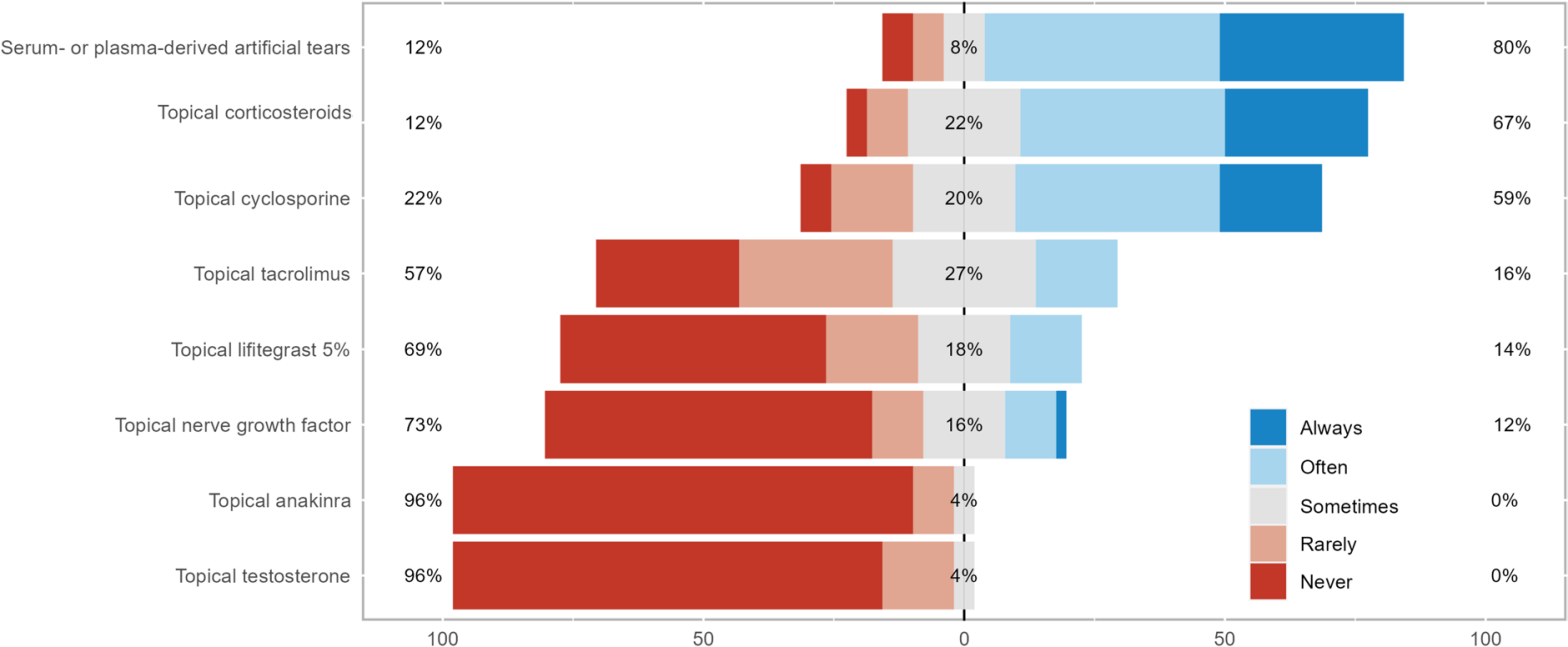
Stacked bar chart of how commonly topical anti-inflammatory and/or neurogenic therapies are used in neuropathic corneal pain

**Fig. 3:**
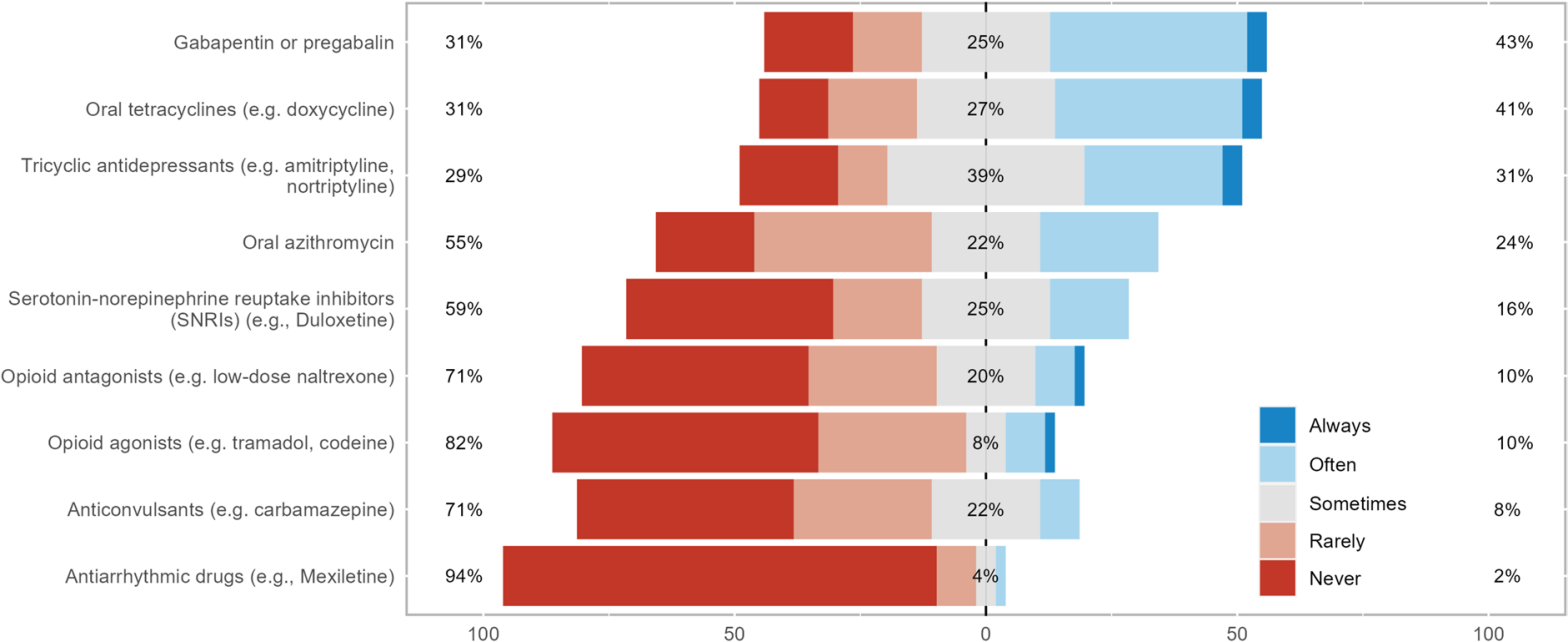
Stacked bar chart of how commonly systemic anti-inflammatory or analgesic therapies are used in neuropathic corneal pain

**Fig. 4:**
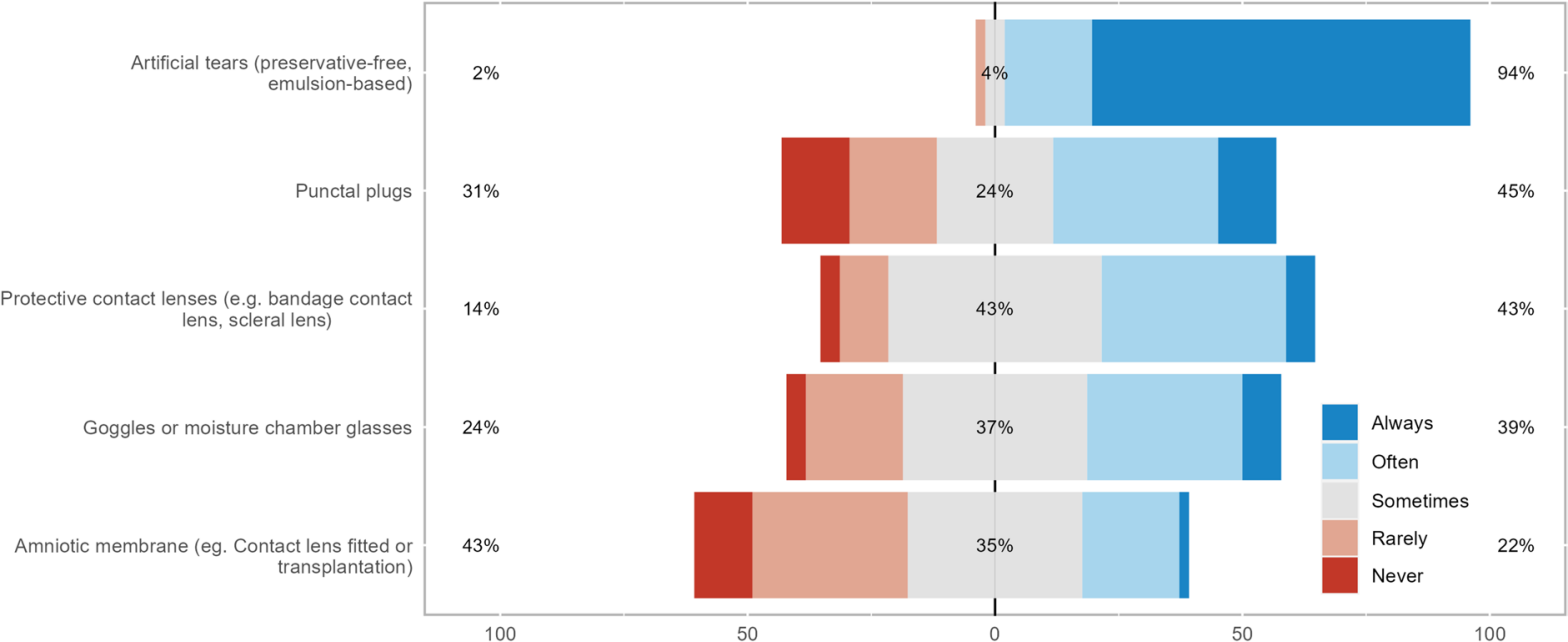
Stacked bar chart of how commonly ocular surface rehabilitation is used in neuropathic corneal pain

**Fig. 5:**
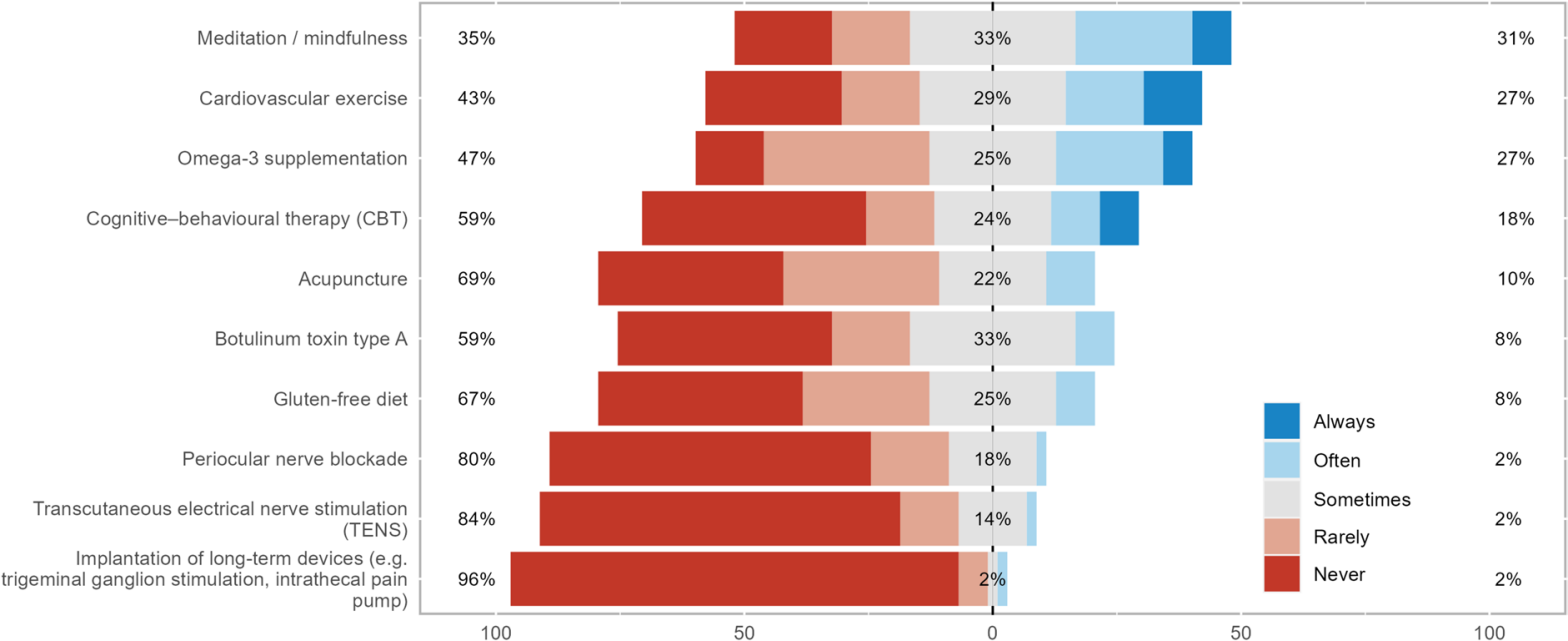
Stacked bar chart of how commonly adjuvant therapies and lifestyle changes are used in neuropathic corneal pain

Treatments for corneal neuropathic pain were categorized into four groups: topical anti-inflammatory and/or neurogenic therapies, systemic anti-inflammatory or analgesic therapies, ocular surface rehabilitation, and adjuvant therapies and lifestyle changes (Figs. 2, 3, 4, and 5). Treatments from across all groups were used, with four drugs having over 50% of respondents stating they used them often or always. These were artificial tears (94%), serum- or plasma-derived tears (80%), topical corticosteroids (67%), and topical cyclosporine (59%). The systemic anti-inflammatory or analgesic therapies and adjuvant therapies and lifestyle changes categories did not have any treatments used often or always by over 50% of respondents, with gabapentin or pregabalin being the most used systemic agents (43%) and meditation or mindfulness being the most commonly recommended lifestyle change (31%).

#### Topical Corticosteroids

Fifteen out of 51 (29%) respondents did not routinely prescribe topical steroids in their management of NCP. They provided 25 reasons explaining this decision. Eleven of 25 (44%) stated it may increase intraocular pressure, seven of 25 (28%) stated it may predispose to ocular infections, four of 25 (16%) stated it may exacerbate ocular surface inflammation if not preservative-free, two of 25 (8%) stated they do not believe it is helpful, with one of 25 (4%) not having access to topical corticosteroids in their practice.

#### Systemic Pharmacotherapy

There was an approximately equal split in the proportion of participants that felt comfortable using systemic pharmacotherapy always or often (*n* = 19, 38%) as opposed to rarely or never (*n* = 19, 38%). Of the 50 respondents that could prescribe systemic pharmacotherapy, 36% (*n* = 18) reported only feeling comfortable prescribing such agents with support from a multidisciplinary team (MDT) or worked with other healthcare professionals to prescribe these.

#### Sub-group Analysis

Further sub-group analysis comparing responses from the United States (US) to Europe (involving 79% of our data) identified that three treatments—topical nerve growth factor, topical lifitegrast and punctal plugs—were used significantly more in the US (*p* = 0.020, *p* < 0.001, *p* = 0.046, respectively). There were no significant differences identified in the investigations performed between the two subgroups.

### MDT

Twenty-four of 51 respondents (47%) worked in a center with an MDT for NCP. Pain management (*n* = 20) and neurology (*n* = 16) were the two most common specialties reportedly involved in MDT discussions. Other specialties included psychology, psychiatry, rheumatology, and nutritionists.

Thirty-five of 51 respondents (69%) routinely referred to other medical specialties for assessment and management, even if the main symptoms are ophthalmic. The most common referral reasons were feeling that the patient would benefit from formal cognitive assessment and/or treatment (42%), if topical treatments were unsuccessful (31%), and if there were doubts regarding the extent of suspected central involvement (23%). Pain specialists (37%), neurology (32%), and psychiatry (17%) were the most common specialties to be referred to.

## Discussion

Our survey provides the first comprehensive overview of the variable clinical assessment and management of NCP worldwide. While primary causes like dry eye syndrome and post-surgical complications (e.g., refractive surgery), are consistently reported, the diversity in current practices highlights a lack of robust evidence to guide clinical decisions [3]. Common treatments such as artificial tears and corticosteroid drops are widely used, yet there is no consensus on their prescribing frequency, strength, and type.

Dieckmann et al. proposed a five-step assessment process for NCP—including symptom questionnaires, functional somatosensory testing, clinical examination, assessment of ocular co-morbidities, and IVCM [3]. Our survey indicates that while these steps are recognized, there is considerable variation in the specific tests applied and their frequency of use. In particular, newer diagnostic tools like the OPAS and NPSI questionnaires, and IVCM, are not broadly used. Additionally, there is considerable variability in multidisciplinary engagement and systemic pharmacotherapy, highlighting the need for collaborative approaches to understand and manage NCP effectively.

Given that minimal or no corneal pathology is often evident on slit-lamp examination [5], new diagnostic criteria and biomarkers for NCP need to be considered. Although there is a general consensus on the importance of esthesiometry, anesthetic challenge test, use of pain questionnaires and the lack of a unified diagnostic criteria results in differing patient cohorts across studies. For instance, one study proposed diagnosing neuropathic pain if at least three of five criteria are met: evidence of somatosensory nervous system damage, minimal corneal damage (Oxford Score ≤ 1), typical neuropathic pain descriptors, abnormal corneal sensitivity, and persistence of symptoms after topical anesthesia [7]. Establishing clear diagnostic and therapeutic targets would facilitate formal, evidence-based diagnosis and investigations into various therapies for NCP, enabling multicenter collaboration and replication of findings.

IVCM, an emerging tool for biomarker detection, can identify changes in the stromal and sub-basal nerves in patients without ocular surface signs [2, 4]. Microneuromas—swelling of injured nerves at their terminal endings—are perhaps the most specific clinical sign demonstrated in NCP. One study reported that IVCM had 100% sensitivity and specificity for detecting microneuromas in NCP [8]. However, another study found no significant difference in microneuroma frequency between patients with dry eye and NCP features versus patients with dry eye without NCP features. These contrasting results may be due to differing populations, the subjective nature of discerning microneuromas, and varying definitions of NCP [9]. Other IVCM findings possibly related to NCP include reduced nerve fiber density, increased beading and branching, and sub-basal nerve tortuosity [5, 8]. These signs warrant further investigation in larger samples and across different NCP causes, such as Sjögren’s syndrome and post-infectious (e.g., herpetic) [5].

In our survey, only 37% (*n* = 19) of our respondents use IVCM routinely for NCP diagnosis, while an additional 39% (*n* = 20) use it when the clinical picture is unclear. Those who rarely or never use IVCM often cite the unavailability of equipment or trained staff as barriers. The utility of IVCM in equivocal cases may need further demonstration before resources are allocated to overcome these limitations. Accessibility of such techniques across different treatment settings should be considered when developing consensus recommendations.

Identifying whether NCP affects the peripheral or central nervous system can help guide treatment decisions and is highly recommended by 77% (*n* = 40) of respondents, with systemic pharmacotherapy particularly used for central NCP [5, 10]. The significance of identifying the primary pain pathways is reflected in our results, with the anesthetic challenge test being the most used investigation. This test involves using a drop of anesthetic that reduces or completely eliminate peripheral neuropathic pain but does not relieve central NCP [5].

Despite this, over 50% of respondents do not use systemic therapy. Limited experimental and observational studies suggest the possible efficacy of systemic pain relief (e.g., gabapentin) in modulating NCP symptoms [11, 12], but high-level evidence is lacking [3]. Furthermore, over a third of respondents reported they would not feel confident prescribing systemic therapy without MDT consultation, mainly due to a lack of experience. Further training for ophthalmologists in systemic therapy could facilitate management.

Ocular pain and surface questionnaires are used by 69% of specialists in our survey, providing insight into patient’s symptoms and quality of life. Although OPAS is validated to measure eye pain severity of any origin, it was less commonly used than the OSDI, a dry-eye questionnaire [3, 5]. Similar trends were also seen with NPSI and NPSI-eye, both validated for neuropathic pain [13, 14], but less commonly used than OSDI, DEQ-5, and OPAS in our survey. It remains unclear how well these scores capture the utility and loss experienced by patients with NCP; further patient-reported outcome research is needed. In research settings, the choice of questionnaire and relevant endpoints should align with regulatory expectations.

Somatosensory testing, particularly corneal esthesiometry, was used by over 70% of respondents. However, interpreting results from this technique is debated: some studies report reduced sensitivity in patients with NCP [4, 5, 15], while others note increased sensitivity [16]. This discrepancy underscores the need for further research to clarify corneal esthesiometry’s role in diagnosing and understanding NCP.

Current literature on NCP treatment often advocates a multimodal approach targeting different pathologies exacerbating the pain. Our results show that the most common treatments are topical agents aiding ocular surface maintenance, with topical artificial tears used by nearly all respondents. These drops help with coexisting dry eye disease and provide short-term symptom improvements [3]. Serum- or plasma-derived tears are also commonly used (80% of respondents), as these contain neurotrophic factors that may reduce pain and help regenerate corneal nerves, as seen on IVCM [17, 18].

Topical anti-inflammatory medicines such as corticosteroids and cyclosporine were used by over 50% of our respondents. Damage to peripheral nerves in NCP leads to peripheral sensitization, as these release pro-inflammatory neuropeptides and trigger inflammatory cytokine release from nearby healthy nerves [3]. These medications are presumed to decrease immune cell recruitment to the cornea, breaking the cycle of chronic inflammation and reducing pain and discomfort [5].

Although our survey did not identify these, several experimental topical therapies have been reported in the literature. Lacosamide, which acts to enhance the slow inactivation of voltage-gated sodium ions, reduced hyper-excitability of cold-sensitive nerve terminals in the cornea in ex vivo models [19, 20]. Topical enkephalin modulators decrease corneal mechanical and chemical hypersensitivity in murine models [20, 21]. Topical transient receptor potential vanilloid-1 (TRPV1) antagonists reduced corneal hypersensitivity in severe dry eye disease mouse models and alleviated post-surgical pain following photorefractive keratectomy in humans [22–24]. Their absence in our respondent’s reports likely reflects the limited current evidence base for these treatments.

Our study had a 34% response rate, consistent with other ophthalmology surveys and general web-based surveys of physicians [25–29]. However, this may introduce sampling bias, potentially limiting the generalizability of our findings. Additionally, 85% of respondents were from North America and Europe, impacting the study’s external validity in other regions globally.

## Conclusions

While the most common causes of NCP are consistent across clinical settings, the variability in its investigation and management reflects a limited understanding of its underlying mechanisms. Most corneal specialists rely on topical treatments such as artificial tears, serum- and plasma-derived tears, and topical corticosteroids for symptomatic relief, with systemic pharmacotherapy used more cautiously. More research and clinical trials are needed to better understand NCP and develop new therapies. Emerging diagnostic tools such as IVCM offer potential insights into NCP’s pathogenesis and therapeutic biomarkers, though their clinical utility and accessibility remain unclear. Furthermore, future research could employ a Delphi method to develop consensus-based questionnaires and guidelines for the assessment and management of NCP. In the meantime, we recommend (A) using questionnaires at initial presentation and follow-up to aid management, assess quality of life impact, monitor disease progression, and evaluate treatment response, and (B) greater multidisciplinary collaboration, particularly involving pain management and neurology specialists, to improve outcomes in this challenging condition.

## Data Availability

The datasets generated during and/or analyzed during the current study are available in Mendeley Data at 10.17632/djfc767d42.1 [30].
